# Correction: Identifying key physiological and clinical factors for traumatic brain injury patient management using network analysis and machine learning

**DOI:** 10.1371/journal.pone.0338553

**Published:** 2025-12-09

**Authors:** The PLOS One Staff

## Notice of Republication

This article was republished on November 18, 2025, to correct errors in the citation. The publisher apologizes for the error. Please download this article again to view the correct version. The originally published, uncorrected article and the republished, corrected articles are provided here for reference.

## Supporting information

S1 File Originally published, uncorrected article. (PDF)

S2 File Republished, corrected article. (PDF)
