# Supplementary figures and images for: Identifying key physiological and clinical factors for traumatic brain injury patient management using network analysis and machine learning
Source: PLoS One. 2025 Jul 28;20(7):e0328870. doi: 10.1371/journal.pone.0328870 (PMC12303317; doi:10.1371/journal.pone.0328870)

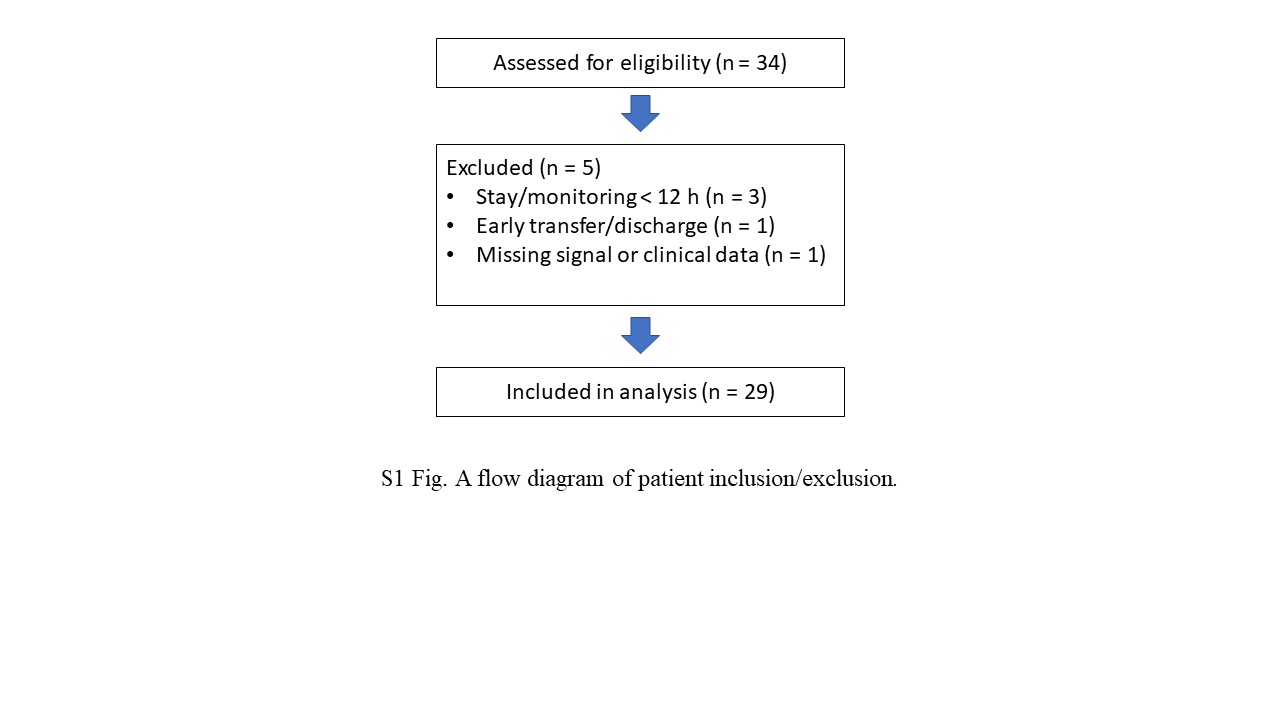

Supplement: S1 Fig — (TIF) [file pone.0328870.s001.tif]

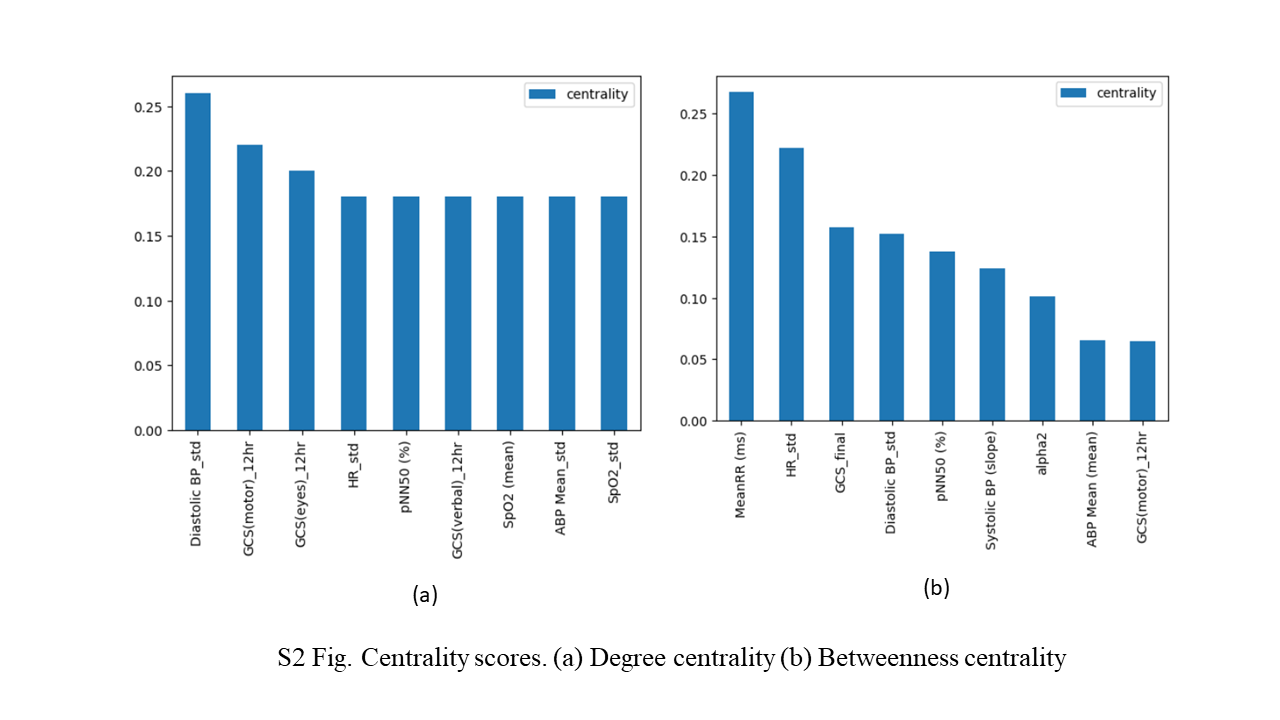

Supplement: S2 Fig — (a) Degree centrality (b) Betweenness centrality. (TIF) [file pone.0328870.s002.tif]

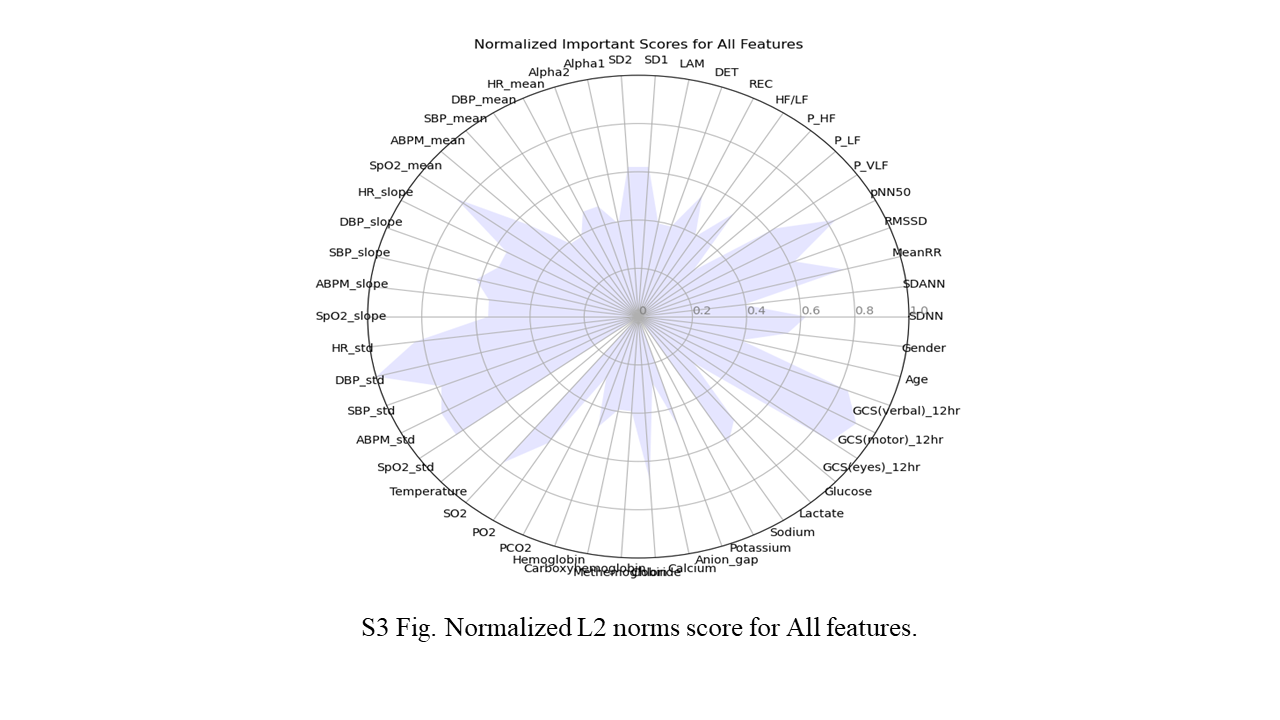

Supplement: S3 Fig — (TIF) [file pone.0328870.s003.tif]

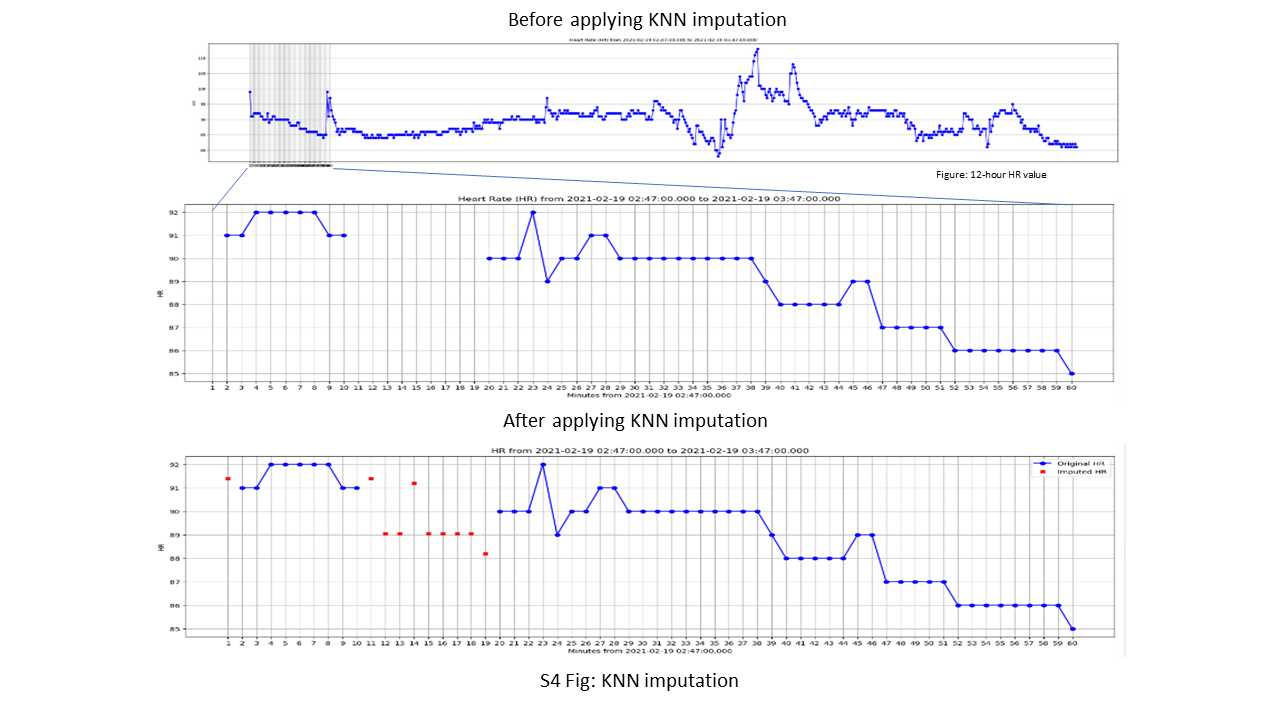

Supplement: S4 Fig — (TIF) [file pone.0328870.s004.tif]
