# Supplementary material for: Identifying key physiological and clinical factors for traumatic brain injury patient management using network analysis and machine learning
Source: PLoS One. 2025 Jul 28;20(7):e0328870. doi: 10.1371/journal.pone.0328870 (PMC12303317; doi:10.1371/journal.pone.0328870)
Supplement: S1 Table — (PDF) [file pone.0328870.s005.pdf]

S1 Table: Baseline patient characteristics

Variables	Total (n = 29)	GCS_final		
		Mild (GCS 13–15, n = 19)	Moderate (GCS 9–12, n = 7)	Severe (GCS 3–8, n = 3)
Age				
< 40 yrs	13 (45%)	9 (47%)	3 (43%)	1 (33%)
40–60 yrs	9 (31%)	7 (37%)	2 (28.5%)	
> 60 yrs	7 (24%)	3 (16%)	2 (28.5%)	2(67%)
GCS_12 hr				
Mild (13–15)	6 (21%)	6 (32%)		
Moderate (GCS 9–12)	4 (14%)	2 (11%)	2 (29%)	
Severe (GCS 3–8)	19 (65%)	11 (57%)	5 (71%)	3 (100%)
Length of Stay in ICU (days)				
Median	57	46	157	54
