# Supplementary material for: Identifying key physiological and clinical factors for traumatic brain injury patient management using network analysis and machine learning
Source: PLoS One. 2025 Jul 28;20(7):e0328870. doi: 10.1371/journal.pone.0328870 (PMC12303317; doi:10.1371/journal.pone.0328870)
Supplement: S2 Table — (PDF) [file pone.0328870.s006.pdf]

S2 Table. Top 20 features ranked by L_2 norm of their node embeddings from the GCN analysis.

Feature	L_2 norm for node embeddings
DBP_std	3.59872
GCS(motor)_12hr	3.30790
GCS(eyes)_12hr	3.15929
pNN50	2.99853
GCS(verbal)_12hr	2.99835
SpO2_std	2.99634
HR_std	2.99462
SpO2_mean	2.99452
ABPM_std	2.98920
MeanRR	2.83177
SO2	2.83015
SBP_std	2.81753
Chloride	2.44761
Sodium	2.23866
SD2	2.23751
SD1	2.23733
P_VLF	2.23638
SDNN	2.23597
RMSSD	2.23566
PO2	2.23123
