# Supplementary material for: Correction: Identifying key physiological and clinical factors for traumatic brain injury patient management using network analysis and machine learning
Source: PLoS One. 2025 Dec 9;20(12):e0338553. doi: 10.1371/journal.pone.0338553 (PMC12688117; doi:10.1371/journal.pone.0338553)
Supplement: S1 File — (PDF) [file pone.0338553.s001.pdf]

PLOS One

RESEARCH ARTICLE

Identifying key physiological and clinical factors for traumatic brain injury patient management using network analysis and machine learning

Hasitha Kuruwita Arachchige[1]*, **Shu Kay Ng**[1], **Alan Wee-Chung Liew**[2], **Brent Richards**[3], **Luke Haseler**[4], **Kuldeep Kumar**[5], **Kelvin Ross**[6], **Ping Zhang**[1]

1 School of Medicine and Dentistry, Griffith University, Queensland, Australia, **2** School of ICT, Griffith University, Queensland, Australia, **3** IntelliHQ, Gold Coast, Australia, **4** Curtin School of Allied Health, Curtin University, Perth, Australia, **5** Bond Business School, Bond University, Gold Coast, Australia, **6** Clinical Data Services, Datarwe, Gold Coast, Australia

* hasitha.kuruwitaarachchige@griffithuni.edu.au

Check for updates

OPEN ACCESS

Citation: Kuruwita Arachchige H, Kay Ng S, Liew AW-C, Richards B, Haseler L, Kumar K, et al. (2025) Identifying key physiological and clinical factors for traumatic brain injury patient management using network analysis and machine learning. PLoS One 20(7): e0328870. https://doi.org/10.1371/journal.pone.0328870

Editor: Eric Anthony Sribnick, Nationwide Children's Hospital, UNITED STATES OF AMERICA

Received: January 7, 2025

Accepted: July 8, 2025

Published: July 28, 2025

Copyright: © 2025 Kuruwita Arachchige et al. This is an open access article distributed under the terms of the Creative Commons Attribution License, which permits unrestricted use, distribution, and reproduction in any medium, provided the original author and source are credited.

Data availability statement: The minute-by-minute physiological recordings and raw ECG spectral data remain potentially re-identifiable and thus fall under the

Abstract

In the intensive care unit (ICU), managing traumatic brain injury (TBI) patients presents significant challenges due to the dynamic interaction between physiological and clinical markers. This study aims to uncover these subtle interconnections and identify the key ICU markers for the timely care of TBI patients using advanced machine-learning techniques. We combined correlation-based network analysis and graph neural network (GNN) techniques to explore relationships among electrocardiography (ECG) features, vital signs, pathology test results, Glasgow Coma Scale (GCS) scores, and demographics from 29 TBI patients admitted to the Gold Coast University Hospital (GCUH). Our findings highlighted that the final GCS index strongly correlated with arterial and diastolic blood pressure variations, patient demographics such as gender and age, and certain heart rate variability (HRV) features. Variability in diastolic blood pressure, GCS, and pNN50 (an HRV measure) demonstrated strong associations with several other physiological and clinical markers during the first 12 hours post-ICU admission. HRV features and variability in physiological signals during the first 12 hours in the ICU are important factors in assessing the severity of TBI patients.

1. Introduction

Traumatic brain injury (TBI) is a major cause of mortality and injury disability worldwide [1]. Managing traumatic brain injuries in intensive care units (ICU) is challenging due to the variety of patient representations, extensive comorbidities, and complications resulting from primary damage. Determining the injury severity and monitoring the patient's progress are complex tasks. Clinicians seek specific markers that can assess patient progress, possible complications, and the relationships between these

restrictions of our Human Research Ethics Committee (HREC) approval. Accordingly, these de-identified datasets are not publicly archived but are available upon reasonable request from Datarwe Pty Ltd (https://datarwe.com/contact-conduct-research-thanks/), subject to a data-use agreement and approved ethics exemption. For all inquiries and to initiate a request, please contact via lucy.shinners@datarwe.com or steve.woodyatt@datarwe.com, +61 418 362 550.

Funding: The author(s) received no specific funding for this work.

Competing interests: The authors have declared that no competing interests exist.

markers and patient outcomes, thereby facilitating informed clinical decision-making. A critical aspect of TBI treatment involves understanding autonomic dysfunction, which can lead to cardiac irregularities, disturbances in regional blood flow, and electrolyte imbalances [2]. Assessing heart rate variability (HRV) along with intracranial pressure (ICP), arterial blood pressure (ABP), oxygen flow, and carbon dioxide levels, plays a vital role in assessing autonomic drive and the physiological status of the brain [2]. However, the specific mechanisms underlying cardiac complications and their relationships with other physiological and respiratory systems are not yet fully understood. Some studies have indirectly explored this relationship or focused on specific aspects, and there is a need for more direct and comprehensive investigations [3].

Heart rate variability, reflecting the variability in the time interval between consecutive heartbeats, serves as an important marker of autonomic nervous system (ANS) activity and has gained attention for its prognostic value in various clinical settings, including intensive care. Recent research has highlighted the predictive ability of HRV for mortality and clinical outcomes in patients with TBI [3], suggesting a complex interplay between autonomic regulation and TBI severity. Given HRV's sensitivity to changes in autonomic tone, it serves as a potential tool for assessing the systemic impact of TBI on patient health, going beyond traditional markers to offer insights into the autonomic dysfunction commonly observed post-injury [4]. HRV has been studied in relation to demographic and clinical factors [5], and its utility as a physiological marker has been demonstrated [6]. Furthermore, Hilz et al. [7] have explored the correlation between the severity of TBI and autonomic impairment observed more than 6 months post-injury using heart rate, blood pressure, and Glasgow Coma Scale (GCS) values. However, the scope of these studies has been limited, as they did not explore the correlation between cardiovascular autonomic parameters and other autonomic parameters. Early work on IMPACT models [8,9] confirmed that GCS admission motor score, tomographic scan characteristics, and age play a crucial role in predicting outcomes in patients with TBI, highlighting the significance of these markers in prognostic assessment. Fluctuations in physiological markers, such as blood pressure and pulse rate, are closely linked to patient outcomes, underscoring the importance of continuous monitoring and targeted interventions to optimize patient outcomes [10]. However, there is still a significant gap in our knowledge regarding how HRV metrics correlate with other physiological markers in TBI patients, particularly during the critical early hours of ICU admission. Most studies have focused solely on HRV or have examined it in limited clinical contexts, neglecting its potential interrelations with other physiological markers that are essential for TBI management [11].

Correlation network analysis (CNA) and graph neural networks (GNNs) are cutting-edge tools in biomedical research that enable the unravelling of complex patterns and correlations across diverse datasets. Correlation-based network analysis explores correlations and uncovers patterns among various data categories and communities. For example, it has been employed to gain insight into the complex interconnections within multi-dimensional biomarker profiles in obesity [12], identify patterns of immune-microbiome interactions [13], and to map comorbidity

relationships through correlation of gene expression data [14]. Similarly, GNNs have been used in medical tasks such as disease prediction, side-effect prediction, and protein interface prediction [15–17]. GNNs demonstrate impressive proficiency in capturing link-type correlations [18,19], including image-guided disease diagnosis [20], which shows potential in aggregating pairwise relations. Moreover, GNNs' ability to effectively process graph-based inputs [20] and their superior performance in network analysis [21] make them a valuable asset in medical research including biomarker analysis [22], biomarker identification [23], learn biomarker interactions to enable breast cancer prognosis [24] and describe the connection between brain regions for neuromodulation in epilepsy [25].

In this pilot study, we employed a data analysis approach integrating CNA and GNNs to examine the complex interrelationships among various markers, including HRV, in ICU-admitted TBI patients. This novel approach in TBI research enables sophisticated analysis of physiological and clinical marker interactions, aiming to understand TBI characteristics and regulatory mechanisms impacting ICU outcomes and inform clinical management. By shedding light on these complex marker interactions, we aim to address a critical gap in existing literature and contribute to the advancement of future research and therapeutic strategies in the treatment of TBI.

2. Materials and methods

2.1. Data collection

This study used data collected from 34 TBI patients who were admitted to the Gold Coast University Hospital (GCUH) in Australia between June 2020 to June 2022. All patient-related data underwent de-identification and was accessible via Datarwe's [26] research platform. Datarwe is a clinical data-as-a-service provider that operates through a collaboration between public and private entities. The data included patient records of electrocardiography (ECG) at 240 Hz, vital sign signals, demographics, pathology test results, and the ICU injury scores (Glasgow Coma Scale). During the early hours after brain injury, the body undergoes various physiological changes, including dynamic changes in brain structure and connectivity [27,28], significant changes in heart rate variability reflecting sympathetic-parasympathetic imbalance [3], and frequent electrolyte imbalances [29]. Early interventions can have a significant impact on patient outcomes and recovery, so we collected the data from the earliest stage after patient admission. However, due to high noise and missing values in the data from the first hour after patient admission, we used data from the 2nd hour until twelve hours after admission. Five patients were excluded due to insufficient data, as their recording periods were less than 12 hours (due either to early discharge, transfer to another facility, or missing data for several hours), and they lacked physiological signals or clinical data. A CONSORT-style flow diagram of patient inclusion/exclusion is provided in S1 Fig. Baseline cohort characteristics are provided in S1 Table.

This study was conducted in accordance with the approved protocol and was exempt from review by the Queensland Health Human Research Ethics Committee (HREC) Australia, as it involved only low-risk, de-identified data (Ref: EX/2022/QGC/86736).

2.2. Study design

Statistical and mathematical methods were used to extract and calculate additional features from the original physiological signals. We performed HRV analysis in time, frequency, and nonlinear domains to extract features from the ECG signal (see Table 1). We then employed a correlation matrix and correlation-based network to reveal hidden relationships, interactions, and dependencies among these fifty-one features. Pearson's correlation coefficient (r), was used to measure the linear relationship between pairs of features, and a threshold of $|r| = 0.5$ was set to determine which correlations were strong enough to be included in the network. The GNN comprised two key components: nodes representing individual feature values and edge connections based on the correlations derived from our analysis. To evaluate the overall connectivity within the network graph, we employed a graph convolutional network (GCN), a type of GNN, to perform unsupervised node embedding. The pipeline of the study methodology is illustrated in Fig 1.

Table 1. Description of features.

HRV features	Vital signs features	Pathology test results	Injury scores	Demographics
SDNN – Measures the standard deviation of normal-to-normal RR for an entire measurement	HR_mean- Mean of heart rate	SO2 – Oxygen saturation level	GCS (eyes)_12hr - Value of Glasgow coma scale eye response value at 12-hour time window	Age
SDANN – Measure the standard deviation of the average normal-to-normal RR interval in all 5 min segments of the entire recordings	HR_std – Standard deviation of heart rate	PO2 – Partial pressure of oxygen level	GCS (motor)_12hr - Value of Glasgow coma scale motor response value at 12-hour time window	Gender
MeanRR – Measure the mean value of RRI for an entire measurement	HR_slope – Slope of heart rate	PCO2 – partial pressure of carbon dioxide level	GCS (verbal)_12hr - Value of Glasgow coma scale verbal response value at 12-hour time window	
RMSSD – Calculation of the square root of the mean squared differences in successive RRIs	DBP_mean – Mean of diastolic blood pressure	Haemoglobin	GCS_final – Final recorded total value of Glasgow coma scale	
pNN50 – Number of interval differences of successive RRI intervals >50 ms divided by the total number of RRI intervals	DBP_std – Standard deviation of diastolic blood pressure	Carboxyhemoglobin		
p_VLF (%) – Power in very low-frequency range (0<= 0.04 Hz)	DBP_slope – Slope of diastolic blood pressure	Methemoglobin		
p_LF (%) – Power in the low-frequency range (0.04–0.15 Hz)	SBP_mean – Mean of systolic blood pressure	Chloride		
p_HF (%) – Power in the high-frequency range (0.15–0.4 Hz)	SBP_std – Standard deviation of systolic blood pressure	Calcium		
HF/LF – Ratio HF/LF	SBP_slope – Slope of systolic blood pressure	Anion_gap		
REC – The percentage of recurrent points in the embedded phase space	ABPM_mean – Mean of arterial blood pressure mean	Potassium		
DET – The ratio of the length of diagonal lines (recurrences) to the length of the whole trajectory	ABPM_std – Standard deviation of arterial blood pressure mean	Sodium		
LAM – The ratio of vertical lines (recurrences) to diagonal lines in the recurrence plot	ABPM_slope – Slope of arterial blood pressure	Lactate		
SD1 – Standard deviation1 – short term variability	SpO2_mean - Mean of saturation of peripheral oxygen	Glucose		
SD2 – Standard deviation2 – long term variability	SpO2_std - Standard deviation of saturation of peripheral oxygen			
Alpha1- Detrended fluctuations alpha 1, measurement of the correlation within the signal	SpO2_slope - Slope of saturation of peripheral oxygen			
Alpha2 – Detrended fluctuations alpha 2 – measurement of the correlation within the signal	Temperature			

https://doi.org/10.1371/journal.pone.0328870.t001

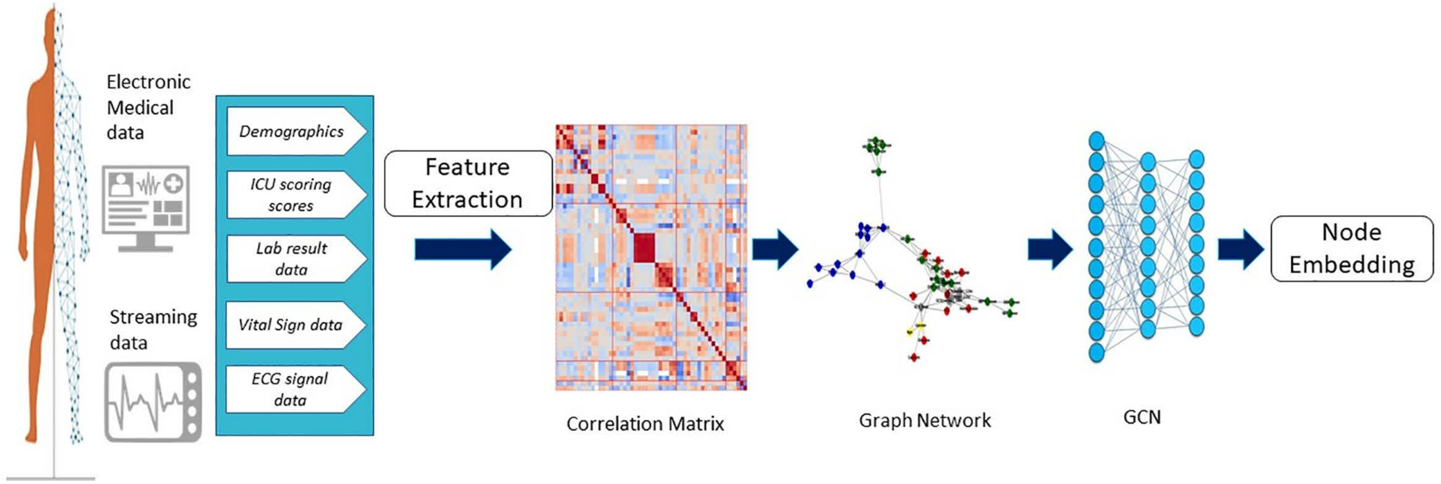

Fig 1. Overview of the study pipeline. Abbreviations: GCN, graph convolutional network.

https://doi.org/10.1371/journal.pone.0328870.g001

2.3. Feature extraction

Heart rate variability measurements were performed in both the time and frequency domains employing the methods described in [30,31]. R-to-R intervals (RRIs) were obtained by detecting R peaks using the Hilbert transform method [32]. Time-domain analysis allows for the extraction of various features from raw RRIs. The Welch method proposed in a previous study [33], was used to quantitatively analyse the power spectral density (PSD) of the RRI across three frequency bands: very low frequency (VLF: 0.005–0.04 Hz), low frequency (LF: 0.04–0.15 Hz), and high frequency (HF: 0.15–0.4 Hz). HRV features were calculated over consecutive hourly windows, and then averaged across the 12 hourly segments to yield one summary value per patient. Nonlinear HRV features were derived based on those proposed in several studies [34,35], including Poincare plots [36], recurrence quantification analysis (RQA) [37], and detrended fluctuation analysis (DFA) [38]. Poincare analysis and RQA are commonly employed to investigate changes in HRV resulting from endurance training, as well as to assess statistical features including standard deviation (SD) and recurrence of patterns [39–41]. DFA is another widely used method that quantifies the scaling properties of ECG data [42–44].

Vital sign signals, including heart rate, diastolic and systolic blood pressure, arterial blood pressure, peripheral oxygen saturation, and respiratory rate, were collected minute-by-minute. The recorded temperature was collected after 12 hours from the time of admission. However, due to ventilator settings' clinical management in the critical initial hours for TBI patients, the respiratory rate was excluded from our analysis, since it was considered a less reliable marker of physiological response. Several patients exhibited gaps of varying length in their minute-by-minute recordings. To account for imputation uncertainty before statistical analysis, missing data were addressed using the k-nearest neighbor (k-NN) imputation method, where the imputed values were typically the mean of k-NN values, following the approach suggested by Zhang et al. [45]. Then signals were transformed into hourly features using statistical analysis, with mean and standard deviation calculated. Additionally, the slope of the best-fit linear regression line for each vital sign was computed over a 12-hour period to interpret trends and fluctuations in the measures. All these derived features were categorised as vital sign features.

Patient age at admission to the ICU and gender were categorised as demographic features. A list of pathological test results was considered as one group of features that were consistently recorded and utilized throughout all analyses. Patient's level of consciousness after brain injury was measured by GCS, which assesses patients based on their ability to perform three aspects of responsiveness: eye-opening, motor, and verbal responses. So, the GCS is further divided into

three parameters: GCS_eyes, GCS_motor, and GCS_verbal. Higher scores indicate better responses. In this study, we utilized the GCS score at discharge (GCS_final) strictly as an in-ICU neurological snapshot to explore its correlation with early ICU biomarkers.

In this study, all physiological markers, clinical markers, HRV features, and derived features from vital signs are collectively referred to as 'features' from here for simplicity and consistency. A detailed list of the features can be found in Table 1.

2.4. Correlation network analysis and graph convolutional network

To reveal the interactions between features and capture complex dependencies in graph-structured data [16,46], we employed CNA and GCN. In our study, we constructed a correlation network with nodes representing features and edges representing correlations between pairs of nodes. Within the network, the length of edges was used to represent the level of correlation between the paired features, with shorter lengths indicating higher correlation coefficients. The node degrees reflected the number of connections/edges a specific feature had with other features. At the same time, betweenness centrality scores measured the number of shortest paths between any two features passing through a node. Nodes with higher centrality scores were regarded as network drivers because of their high interconnectivity.

Centrality indices were used to quantify each node's interconnectedness within the network [47], rather than its direct association with the clinical endpoint. In this study, degree centrality, which measures the importance or influence of a node within a network, was normalized by the maximum possible number of edges (n-1). Similarly, betweenness centrality was normalized by the total number of possible pairs of nodes, calculated as [(n-1) (n-2)/ 2], where n represents the number of nodes in the network [48]. Additionally, network density values were utilised to assess the relationships between different subgroups of features, which are HRV features, vital sign features, pathology test results, injury scores (GCS), and demographic data.

GCNs comprise multiple layers that aggregate information from neighboring nodes and update nodes based on their characteristics and graphical structure. In this study, unsupervised GCN was applied with two graph-convolutional layers to identify the most influential features in TBI correlation network. A grid search was performed over hidden dimensions (32, 64, 128, 256) and learning rates (0.001, 0.01, 0.1), training each configuration for 1000 epochs to minimize the mean squared error (MSE) between the model output and the original node features. Adam optimizer was utilised, and the optimal parameters (hidden dimension = 256 and learning rate = 0.01) were chosen based on the lowest validation MSE. The final model was then trained for 10,000 epochs to ensure convergence. The relevance of each feature was determined by computing the L2 norms [49] of the embedding vectors, with higher values indicating greater significance in the correlation network. To gain a holistic understanding, these scores were normalized across all features. However, these scores only suggest a high level of connectivity and do not directly imply causal importance with respect to clinical outcomes.

The entire feature calculation, correlation analysis, and graph analysis were conducted using Python on the AWS cloud platform. NetworkX [48] and Pyvis [50] were utilized to visualize the correlation network, while PyTorch geometry [51] was utilized for GCN training and analysis.

3. Results

The initial analysis revealed a significant correlation (ρ-value < 0.05) between various features within individual feature groups and across different groups, indicating significant interplay among them (Fig 2). We found a significant positive correlation between HRV features (SDNN, MeanRR, RMSSD, pNN50, SD1, and SD2) and mean calculation of specific vital signs, SBP_mean, SpO2_mean, and slope values (HR_slope, SBP_slope, DBP_slope, ABPM_slope and SpO2_slope). MeanRR value was positively correlated with SBP_slope, while the SpO2_slope for oxygen saturation was positively correlated with several HRV features. Furthermore, there was a strong positive correlation among vital sign slope values (r > 0.94).

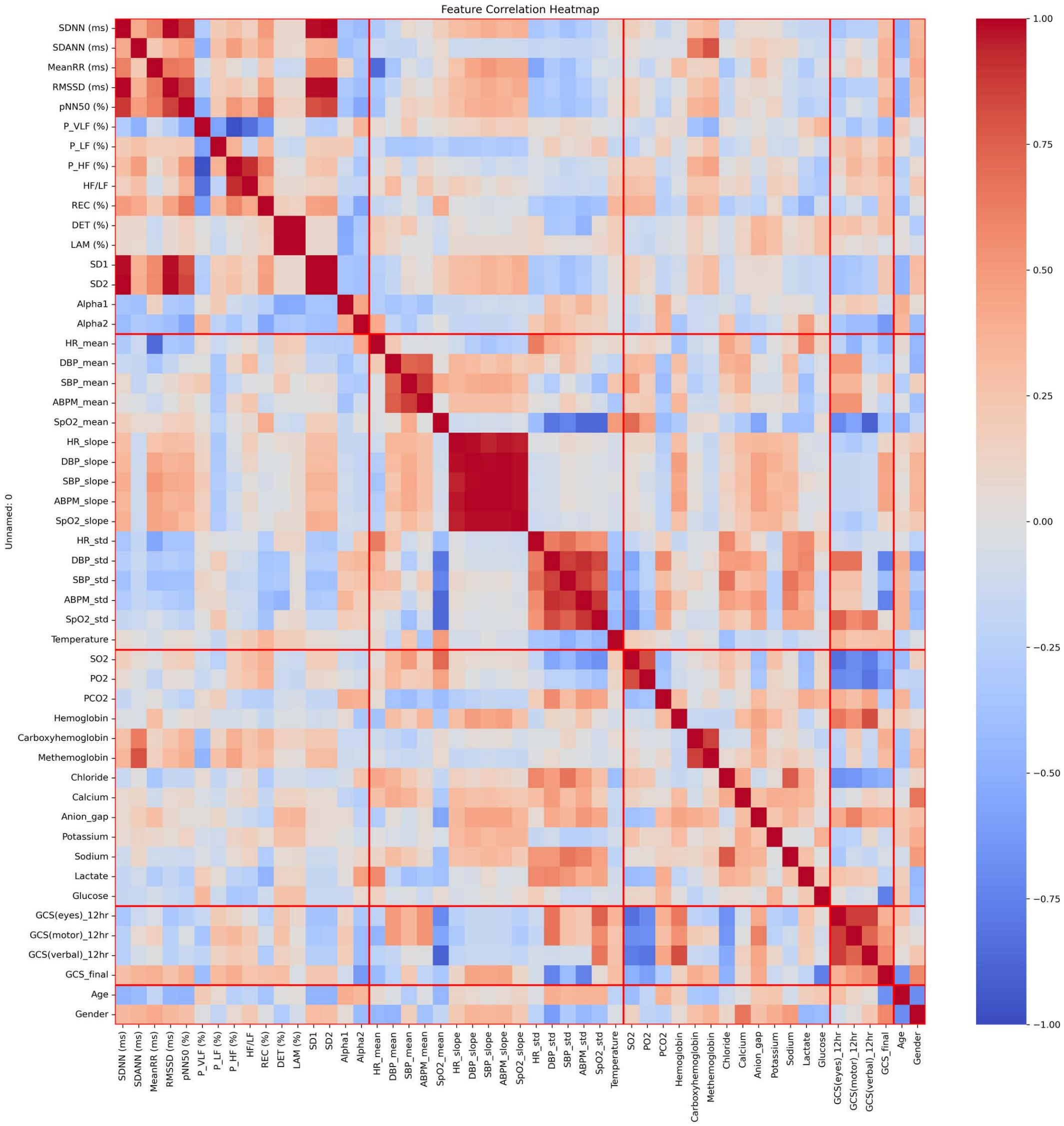

Fig 2. Pairwise correlation heatmaps of the 51 correlated features in five groups (HRV features, vital signs features, pathology test results, injury scores (GCS) and demographics). Abbreviations: SDNN, standard deviation of RR; SDANN, standard deviation of the average RR; MeanRR, mean value of RR; RMSSD, square root of mean squared differences of RR; pNN50, number of interval differences of successive RRI intervals >50 ms divided by the total RRI's; P_VLF, Power in very low-frequency range; P_LF, Power in the low-frequency range; P_HF, Power in the high-frequency range; HF/LF, Ratio HF/LF; REC, percentage of recurrent points; DET, ratio of the length of diagonal lines; LAM, ratio of vertical lines (recurrence plot); SD1, Short term variability; SD2, Long term variability; Alpha1 and Alpha2- correlation within the signal (Detrended fluctuations); HR, heart rate; DBP, diastolic blood pressure; SBP, systolic blood pressure; ABPM, arterial blood pressure mean; SpO2, peripheral oxygen saturation; SO2, oxygen saturation level; PO2, partial pressure of oxygen saturation; PCO2, partial pressure of carbon dioxide level; GCS(eyes)_12hr, Glasgow coma scale eye response after 12 hours from admission; GCS(motor)_12hr, Glasgow coma scale motor response after 12 hours from admission; GCS_12hr (verbal), Glasgow coma scale verbal response after 12 hours from admission; GCS_final, final recorded total value of Glasgow coma scale.

https://doi.org/10.1371/journal.pone.0328870.g002

The GCS injury scores at 12 hours showed a strong correlation with vital sign features, including SpO2_std, which was correlated with GCS (eyes, verbal, and motor) (r = 0.75, 0.66, 0.58, respectively), while SpO2_mean had a high negative correlation with GCS (eyes, verbal) (r ≥ −0.71). The final GCS value demonstrated a substantial connection with HRV feature Alpha2 (r = −0.62), DBP_std (r = −0.74), and ABPM_std (r = −0.74). Age and final GCS injury score had a fairly strong

negative correlation (r = −0.67), whereas male patients were higher than female patients. A strong negative correlation was observed between GCS injury scores at the 12-hour post-admission (eyes, motor, and verbal) and various pathology test results, such as the SO2, PO2, and Chloride values, with higher correlation coefficients of −0.7, −0.68, and −0.53 respectively. The correlation network (Fig 3a) visually depicts the complex interconnections between HRV features, vital signs features, pathology test results, injury scores (GCS), and demographic features (with correlation magnitude ≥ 0.5). The DBP_std exhibited the highest normalized degree centrality (0.25), indicating that it has the most connections to other features, and GCS motor and verbal response scores had high degree centrality of 0.22 and 0.18 respectively (see S3a Fig). HRV features, including MeanRR, pNN50, and Alpha2, demonstrated high betweenness centrality (see S3b Fig). The relationship between features from different groups was visually represented by a single line connecting the two groups (Fig 3b). Group-wise, GCS injury score and pathology test results showed high connectivity and a strong correlation was observed between the GCS scores, demographics, and vital signs features. However, there was no noticeable link between the HRV features and demographic groups.

Fig 4 shows the normalized importance scores of the top 20 features from the GCN analysis, providing a clear visualization of their relative significance within the dataset (see S2 Table for the corresponding L_2 norms values). The standard deviation of diastolic blood pressure demonstrated the highest connectivity, followed closely by GCS injury scores including GCS (motor), GCS (eyes), and GCS (verbal). Among these features, HRV features, including pNN50 and MeanRR, exhibit considerable importance in this correlation network. Moreover, the standard deviations of SpO2, heart rate, arterial blood pressure, and systolic blood pressure were identified as significant vital sign features. Furthermore, several other HRV features, including SD2, SDNN, RMSSD, and SD1, showed a high level of connectivity, indicating their importance as clinical measures. Normalized scores for all features are presented in the S3 Fig.

4. Discussion

Our study examined the inter-relationship among early ICU data during the first 12 hours of ICU admission in 29 TBI patients by combining CAN and GCN. Prior works have leveraged dynamic and data-driven prognostic or clustering ML over broader time-frames or intake/outcome domains rather than focusing on early window. For example, Tritt et al. [52] developed an interpretable prognostic model utilising non-negative matrix factorisation and sparse canonical correlation analysis. In another study [53], a token-embedding encoder plus dynamic recurrent neural network-based analyses of clinical course contributions were performed to predict six-month Glasgow outcome scale extended score (GOSE) outcome. Akerlund et al. [54], performed clustering analysis of disease trajectories over the first week after admission, identifying glucose variability and serial biomarker panels as key trajectory descriptors. However, these works have not examined the inter-relationships among a comprehensive set of clinical and physiological features at a critical time point immediately after ICU admission. To fulfill our aim, we constructed a correlation network and then applied an GCN to identify hub features and models that may enable more timely, targeted interventions.

Our main findings demonstrated a relationship between HRV features, such as SDNN and RMSSD measures, and the linear trends of heart rate, blood pressure, and SpO2, suggesting a more responsive autonomic nervous response regarding cardiovascular parameters. These findings are consistent with prior research that highlights the link between autonomic nervous system activity and cardiovascular health [55]. GCS components play a central role in this network, suggesting their influence on various parameters. The final GCS score was treated as an ordinal value for analysis. The observed linear correlation between the final GCS injury score and other features may shed light on how the overall neurological outcome and level of consciousness in patients relate to other clinical measures. However, it is important to note that this correlation analysis does not fully recognize the various aspects of clinical responsiveness. Correlation examination revealed that gender and age were associated with several physiological and clinical markers, including HRV, blood pressure, and GCS values. Notably, there was a negative correlation between age and the final GCS score, implying that older patients tend to exhibit lower final GCS scores, which may indicate poorer sedation levels or neurological outcomes.

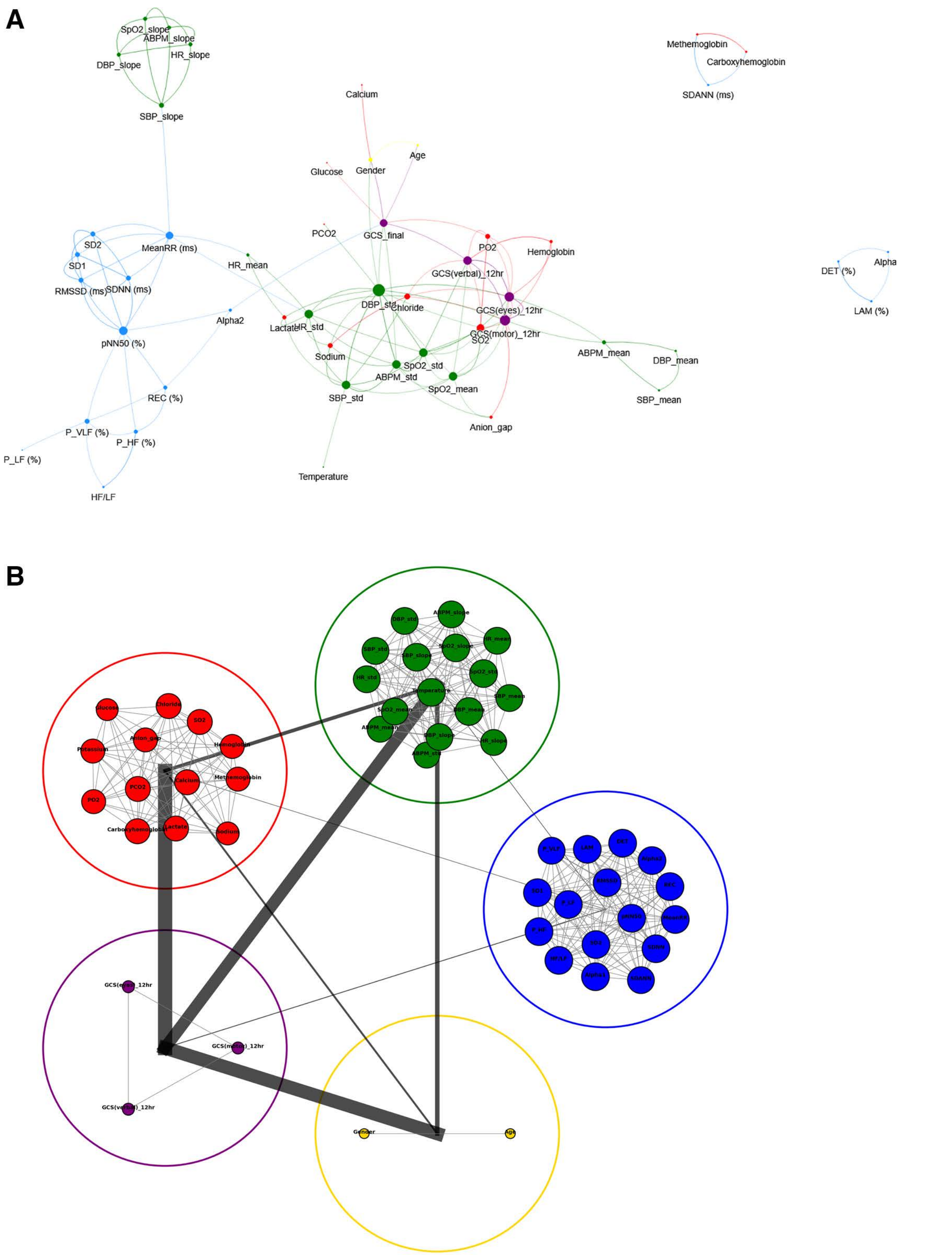

Fig 3. Relationship between physiological/clinical features (a) Witcher Network: Relationship between features and the correlation threshold was 0.5. (b) Relationship between the feature groups. Abbreviations: SDNN, standard deviation of RR; SDANN, standard deviation of the average RR; MeanRR, mean value of RR; RMSSD, square root of mean squared differences of RR; pNN50, number of interval differences of successive RRI intervals >50 ms divided by the total RRI's; P_VLF, Power in very low-frequency range; P_LF, Power in the low-frequency range; P_HF, Power in the high-frequency range; HF/LF, Ratio HF/LF; REC, percentage of recurrent points; DET, ratio of the length of diagonal lines; LAM, ratio of vertical lines

(recurrence plot); SD1, Short term variability; SD2, Long term variability; Alpha1 and Alpha2- correlation within the signal (Detrended fluctuations); HR, heart rate; DBP, diastolic blood pressure; SBP, systolic blood pressure; ABPM, arterial blood pressure mean; SpO2, peripheral oxygen saturation; SO2, oxygen saturation level; PO2, partial pressure of oxygen saturation; PCO2, partial pressure of carbon dioxide level; GCS(eyes)_12hr, Glasgow coma scale eye response after 12 hours from admission; GCS(motor)_12hr, Glasgow coma scale motor response after 12 hours from admission; GCS_12hr (verbal), Glasgow coma scale verbal response after 12 hours from admission; GCS_final, final recorded total value of Glasgow coma scale.

https://doi.org/10.1371/journal.pone.0328870.g003

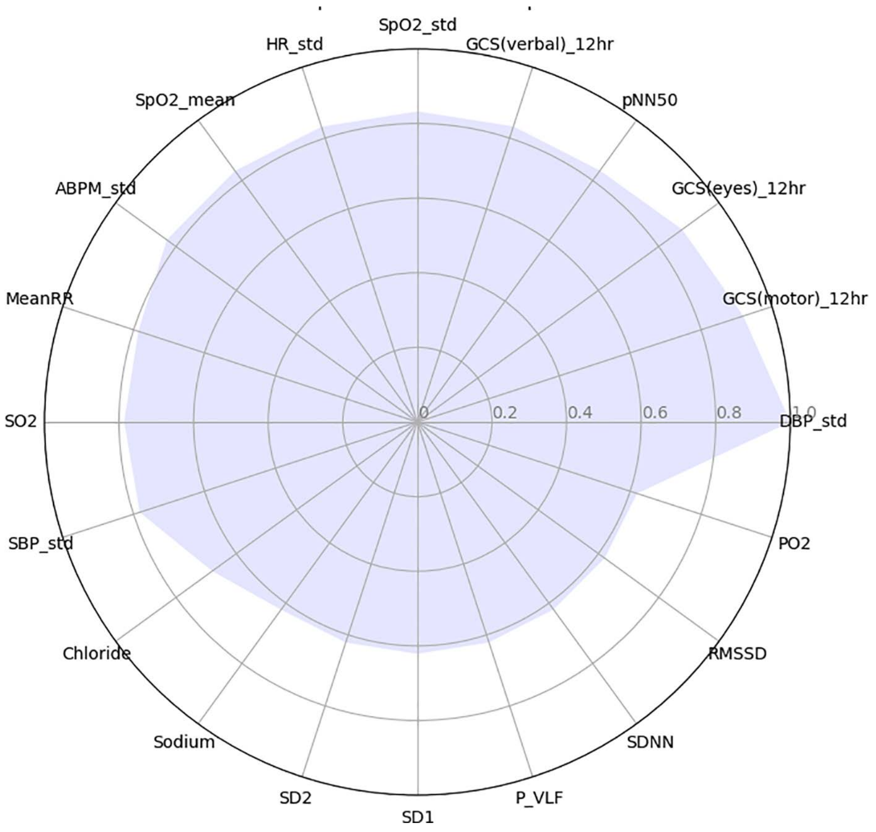

Fig 4. Twenty features with the highest significance scores from GCN analysis. Abbreviations: SDNN, standard deviation of RR; MeanRR, mean value of RR; pNN50, number of interval differences of successive RRI intervals >50 ms divided by the total RRI's; P_VLF, Power in very low-frequency range; SD1, Short term variability; SD2, Long term variability; HR, heart rate; DBP, diastolic blood pressure; SBP, systolic blood pressure; ABPM, arterial blood pressure mean; SpO2, peripheral oxygen saturation; SO2, oxygen saturation level; PO2, partial pressure of oxygen saturation; GCS(eyes)_12hr, Glasgow coma scale eye response after 12 hours from admission; GCS(motor)_12hr, Glasgow coma scale motor response after 12 hours from admission; GCS_12hr (verbal), Glasgow coma scale verbal response after 12 hours from admission.

https://doi.org/10.1371/journal.pone.0328870.g004

This finding is aligned with existing literature [56,57], which highlights the connection between age and the severity of traumatic brain injury, suggesting that age could impact neurological outcomes beyond a straightforward linear relationship.

Moreover, the inverse relationship between GCS eye scores and age reveals age-specific nuances in neurological responses, consistent with existing literature indicating age's role as a modulator of TBI severity. The study [58] highlighted that TBI is less prevalent in females, who exhibited a higher mortality rate and a higher percentage of abnormal findings compared to males. Our findings are consistent with prior reports, suggesting a substantial link between gender and specific HRV measures and several vital sign features. Female patients had lower final GCS scores than male patients, indicating a lower level of injury severity. Some measures, such as SO2 and PCO2, may reflect both clinical

management strategies and the physiological state of the patient. The correlation between SO2 and GCS severity ratings, especially at the 12-hour mark, may suggest a link with neurological recovery, but it should be interpreted with caution as it may also reflect clinical management. The strong correlation between GCS motor response and vital sign statistics underscores the potential interplay between neurological responsiveness and cardiovascular dynamics, which can have significant clinical implications. It suggests that neurological functioning influences cardiovascular health in critically ill patients. The final GCS score showed a strong relationship with Alpha2 (HRV features), variations in diastolic blood pressure, and arterial blood pressure. These results highlight the intricate interactions among circulatory dynamics, autonomic control, and demographic factors that influence the neurological prognosis of patients with TBI in their initial hours in the ICU. The notable betweenness centrality scores of HRV features, such as MeanRR, pNN50, and Alpha2, reveal their influential role in mediating interactions among all features.

Identifying key factors is essential for medical research and clinical practice, and our study revealed that variations in diastolic blood pressure serve as the primary connectivity factor, indicating acute autonomic dysregulation. Although GCS components have traditionally been paramount in assessing neurological functions, they merely represent a patient's condition and injury severity. Multiple HRV features, such as pNN50, MeanRR, VLF, SD1, RMSSD, and SD2, have been proven to be effective early predictors of patient trajectory, consistent with results reported in prior work [59]. Our results emphasize the importance of monitoring physiological variations in TBI, aligning with Lund et al. (2016) findings [11] that variability in physiological signals independently predicts patient severity. Continuous real-time monitoring of these parameters is already available in the ICU through bedside monitors. These data could be harnessed to generate an early warning alert to reassess sedation levels, fluid balance, or optimize autonomic stability. Furthermore, our results emphasize the importance of conventional health indicators such as chloride and sodium levels. These findings suggest a complex interplay among various health metrics in determining patient outcomes and provide valuable insights for health-related research. The application of CAN analysis and GCN for node embedding implies real-world value in clinical prognosis and personalized patient management. High correlation coefficients reveal potential interactions between markers, while GCN's ability to discern complex patterns underscores the importance of a holistic approach to patient assessment and care. The evaluation of physiological and clinical markers sheds light on the complexity of assessing TBI patients.

We acknowledge several limitations of this pilot study. First, our single-centre cohort, particularly the small sample size, may limit our ability to detect more minor correlation effects and raise the possibility of selection bias. We employed an exploratory approach and utilised high correlation coefficient cut-offs, rather than p-values, to define important connections in the correlation network, addressing the constraints of a small sample size. In the future, larger studies will apply formal multiple-comparison corrections (e.g., FDR) to define significant correlations. Second, our data set did not include ICP or cerebral perfusion pressure (CPP) measurements, which are critical for understanding the mechanistic link between HRV and neurological status. Third, other key interventions (e.g., vasoactive medication, ventilation settings, fluid management) were not recorded and therefore represent unmeasured confounders that could influence observed markers. Future work will validate these findings in larger, multicentre cohorts, capture relevant treatment variables, additionally incorporate ICP/CPP data, and leverage time-series feature modelling.

5. Conclusions

In conclusion, this study has thoroughly examined the intricate relationships among various physiologic and clinical markers by employing an innovative approach that involves analysing a comprehensive health dataset during the first 12 hours of ICU admission. The finding of this study shed light on the complex interplay between demographic factors, such as age and gender, and their influence on various physiological and clinical parameters. Notably, variation in diastolic blood pressure has emerged as a key indicator/critical physiological marker in patients with TBI, while GCS components remain fundamental in assessing neurological function. Moreover, HRV demonstrates a significant correlation with vital sign statistics and GCS severity scores, which can underscore the potential of HRV as a valuable marker for assessing severity

and predicting patient outcomes utilised to evaluate autonomic control in patients with TBI. Overall, this study reaffirms the clinical significance of physiological measures.

Supporting information

S1 Fig. A flow diagram of patient inclusion/exclusion.
(TIF)

S2 Fig. Centrality scores. (a) Degree centrality (b) Betweenness centrality.
(TIF)

S3 Fig. Normalize L2 norms score for all features.
(TIF)

S4 Fig. KNN imputation.
(TIF)

S1 Table. Baseline patient characteristics.
(PDF)

S2 Table. Top features ranked by L2 norm of their node embeddings from the GCN analysis.
(PDF)

Author contributions

Conceptualization: Hasitha Kuruwita Arachchige, Ping Zhang.

Data curation: Hasitha Kuruwita Arachchige, Brent Richards, Kelvin Ross.

Formal analysis: Hasitha Kuruwita Arachchige.

Funding acquisition: Kelvin Ross.

Investigation: Hasitha Kuruwita Arachchige.

Methodology: Hasitha Kuruwita Arachchige, Ping Zhang.

Resources: Kelvin Ross.

Software: Hasitha Kuruwita Arachchige.

Supervision: Shu Kay Ng, Alan Wee-Chung Liew, Ping Zhang.

Validation: Brent Richards, Luke Haseler.

Visualization: Hasitha Kuruwita Arachchige.

Writing – original draft: Hasitha Kuruwita Arachchige.

Writing – review & editing: Shu Kay Ng, Alan Wee-Chung Liew, Brent Richards, Luke Haseler, Kuldeep Kumar, Kelvin Ross, Ping Zhang.

References

1. Silver JM, McAllister TW, Arciniegas DB. Textbook of traumatic brain injury. American Psychiatric Pub; 2018.
2. Khalid F, Yang GL, McGuire JL, Robson MJ, Foreman B, Ngwenya LB, et al. Autonomic dysfunction following traumatic brain injury: translational insights. Neurosurg Focus. 2019;47(5):E8. https://doi.org/10.3171/2019.8.FOCUS19517 PMID: 31675718
3. Zhang P, Roberts T, Richards B, Haseler LJ. Utilizing heart rate variability to predict ICU patient outcome in traumatic brain injury. BMC Bioinform. 2020;21(Suppl 17):481. https://doi.org/10.1186/s12859-020-03814-w PMID: 33308142

4. Minassian A, Geyer MA, Baker DG, Nievergelt CM, O'Connor DT, Risbrough VB, et al. Heart rate variability characteristics in a large group of active-duty marines and relationship to posttraumatic stress. Psychosom Med. 2014;76(4):292–301. https://doi.org/10.1097/PSY.0000000000000056 PMID: 24804881
5. Linder JE, Kraft DC, Mohamed Y, Lu Z, Heil L, Tollefson S, et al. Human rhinovirus C: Age, season, and lower respiratory illness over the past 3 decades. J Allergy Clin Immunol. 2013;131(1):69–77.e1-6. https://doi.org/10.1016/j.jaci.2012.09.033 PMID: 23146382
6. Nabasny A, Rabinowitz A, Wright B, Wang J, Preminger S, Terhorst L, et al. Neurobehavioral symptoms and heart rate variability: feasibility of remote collection using mobile health technology. J Head Trauma Rehabil. 2022;37(3):178–88. https://doi.org/10.1097/HTR.0000000000000764 PMID: 35125433
7. Hilz MJ, Wang R, Markus J, Ammon F, Hösl KM, Flanagan SR, et al. Severity of traumatic brain injury correlates with long-term cardiovascular autonomic dysfunction. J Neurol. 2017;264(9):1956–67. https://doi.org/10.1007/s00415-017-8581-1 PMID: 28770375
8. Kulesza B, Nogalski A, Kulesza T, Prystupa A. Prognostic factors in traumatic brain injury and their association with outcome. J Pre-Clin Clin Res. 2015;9(2).
9. Zeiler FA, Donnelly J, Calviello L, Smielewski P, Menon DK, Czosnyka M. Pressure autoregulation measurement techniques in adult traumatic brain injury, Part II: a scoping review of continuous methods. J Neurotrauma. 2017;34(23):3224–37. https://doi.org/10.1089/neu.2017.5086 PMID: 28699412
10. Murray GD, Butcher I, McHugh GS, Lu J, Mushkudiani NA, Maas AIR, et al. Multivariable prognostic analysis in traumatic brain injury: results from the IMPACT study. J Neurotrauma. 2007;24(2):329–37. https://doi.org/10.1089/neu.2006.0035 PMID: 17375997
11. Lund SB, Gjeilo KH, Moen KG, Schirmer-Mikalsen K, Skandsen T, Vik A. Moderate traumatic brain injury, acute phase course and deviations in physiological variables: an observational study. Scand J Trauma Resusc Emerg Med. 2016;24:77. https://doi.org/10.1186/s13049-016-0269-5 PMID: 27216804
12. Chen P-Y, Cripps AW, West NP, Cox AJ, Zhang P. A correlation-based network for biomarker discovery in obesity with metabolic syndrome. BMC Bioinform. 2019;20(Suppl 6):477. https://doi.org/10.1186/s12859-019-3064-2 PMID: 31823713
13. Brisson V, Schmidt J, Northen TR, Vogel JP, Gaudin A. A new method to correct for habitat filtering in microbial correlation networks. Front Microbiol. 2019;10:585. https://doi.org/10.3389/fmicb.2019.00585 PMID: 30949160
14. Moni MA, Liò P. Network-based analysis of comorbidities risk during an infection: SARS and HIV case studies. BMC Bioinform. 2014;15(1):333. https://doi.org/10.1186/1471-2105-15-333 PMID: 25344230
15. Parisot S, Ktena SI, Ferrante E, Lee M, Guerrero R, Glocker B, et al. Disease prediction using graph convolutional networks: application to Autism Spectrum Disorder and Alzheimer's disease. Med Image Anal. 2018;48:117–30. https://doi.org/10.1016/j.media.2018.06.001 PMID: 29890408
16. Zhang X-M, Liang L, Liu L, Tang M-J. Graph neural networks and their current applications in bioinformatics. Front Genet. 2021;12:690049. https://doi.org/10.3389/fgene.2021.690049 PMID: 34394185
17. Zhang L, Zhao Y, Che T, Li S, Wang X. Graph neural networks for image-guided disease diagnosis: a review. iRADIOLOGY. 2023;1(2):151–66. https://doi.org/10.1002/ird3.20
18. Ibrahim AA, Mohammed TA, Dara ON. Predicting Big data Drug Interactions and associated side effects by Using Artificial Neural Networks (ANN) over Traditional Graph Convolutional Networks (GCNs). Research Square Platform LLC; 2024. https://doi.org/10.21203/rs.3.rs-3997856/v1
19. Ma T, Shang J, Xiao C, Sun J. Genn: predicting correlated drug-drug interactions with graph energy neural networks. arXiv preprint. 2019. https://doi.org/10.48550/arXiv.1910.02107
20. Xiao S, Wang S, Dai Y, Guo W. Graph neural networks in node classification: survey and evaluation. Mach Vis Appl. 2021;33(1). https://doi.org/10.1007/s00138-021-01251-0
21. Wang X, Ji H, Shi C, Wang B, Ye Y, Cui P, et al. Heterogeneous Graph Attention Network. In: The World Wide Web Conference. 2019. pp. 2022–32. https://doi.org/10.1145/3308558.3313562
22. Li X, Zhou Y, Dvornek NC, Zhang M, Zhuang J, Ventola P, et al. Pooling regularized graph neural network for fMRI biomarker analysis. Med Image Comput Comput Assist Interv. 2020;12267:625–35. https://doi.org/10.1007/978-3-030-59728-3_61 PMID: 33043324
23. Pan S, Jiang X, Zhang K. WSGMB: weight signed graph neural network for microbial biomarker identification. Brief Bioinform. 2023;25(1):bbad448. https://doi.org/10.1093/bib/bbad448 PMID: 38084923
24. Qiu L, Kang D, Wang C, Guo W, Fu F, Wu Q, et al. Intratumor graph neural network recovers hidden prognostic value of multi-biomarker spatial heterogeneity. Nat Commun. 2022;13(1):4250. https://doi.org/10.1038/s41467-022-31771-w PMID: 35869055
25. Vetkas A, Germann J, Elias G, Loh A, Boutet A, Yamamoto K, et al. Identifying the neural network for neuromodulation in epilepsy through connectomics and graphs. Brain Commun. 2022;4(3):fcac092. https://doi.org/10.1093/braincomms/fcac092 PMID: 35611305
26. "Datarwe," [Online]. [cited 2023 May 12]. Available from: https://datarwe.com/
27. Oertel M, Kelly DF, McArthur D, Boscardin WJ, Glenn TC, Lee JH, et al. Progressive hemorrhage after head trauma: predictors and consequences of the evolving injury. J Neurosurg. 2002;96(1):109–16. https://doi.org/10.3171/jns.2002.96.1.0109 PMID: 11794591

28. Shenton ME, Hamoda HM, Schneiderman JS, Bouix S, Pasternak O, Rathi Y, et al. A review of magnetic resonance imaging and diffusion tensor imaging findings in mild traumatic brain injury. Brain Imaging Behav. 2012;6(2):137–92. https://doi.org/10.1007/s11682-012-9156-5 PMID: 22438191
29. Dey S, Kumar R, Tarat A. Evaluation of electrolyte imbalance in patients with traumatic brain injury admitted in the central ICU of a tertiary care centre: a prospective observational study. Cureus. 2021;13(8):e17517. https://doi.org/10.7759/cureus.17517 PMID: 34595082
30. Tapanainen JM, Seppänen T, Laukkanen R, Loimaala A, Huikuri HV. Significance of the accuracy of RR interval detection for the analysis of new dynamic measures of heart rate variability. Ann Noninvasive Electrocardiol. 1999;4(1):10–7. https://doi.org/10.1111/j.1542-474x.1999.tb00359.x
31. Gula LJ, Krahn AD, Skanes A, Ferguson KA, George C, Yee R, et al. Heart rate variability in obstructive sleep apnea: a prospective study and frequency domain analysis. Ann Noninvasive Electrocardiol. 2003;8(2):144–9. https://doi.org/10.1046/j.1542-474x.2003.08209.x PMID: 12848796
32. Sharma H, Sharma KK. ECG-derived respiration based on iterated Hilbert transform and Hilbert vibration decomposition. Australas Phys Eng Sci Med. 2018;41(2):429–43. https://doi.org/10.1007/s13246-018-0640-0 PMID: 29667117
33. Welch P. The use of fast Fourier transform for the estimation of power spectra: a method based on time averaging over short, modified periodograms. IEEE Trans Audio Electroacoust. 1967;15(2):70–3. https://doi.org/10.1109/tau.1967.1161901
34. Voss A, Kurths J, Kleiner HJ, Witt A, Wessel N. Improved analysis of heart rate variability by methods of nonlinear dynamics. J Electrocardiol. 1995;28 Suppl:81–8. https://doi.org/10.1016/s0022-0736(95)80021-2 PMID: 8656135
35. Guzzetti S, Signorini MG, Cogliati C, Mezzetti S, Porta A, Cerutti S, et al. Non-linear dynamics and chaotic indices in heart rate variability of normal subjects and heart-transplanted patients. Cardiovas Res. 1996;31(3):441–6. https://doi.org/10.1016/s0008-6363(95)00159-x
36. Hoshi RA, Pastre CM, Vanderlei LCM, Godoy MF. Poincaré plot indexes of heart rate variability: relationships with other nonlinear variables. Auton Neurosci. 2013;177(2):271–4. https://doi.org/10.1016/j.autneu.2013.05.004 PMID: 23755947
37. Zbilut JP, Thomasson N, Webber CL. Recurrence quantification analysis as a tool for nonlinear exploration of nonstationary cardiac signals. Med Eng Phys. 2002;24(1):53–60. https://doi.org/10.1016/s1350-4533(01)00112-6 PMID: 11891140
38. Penzel T, Kantelhardt JW, Grote L, Peter J-H, Bunde A. Comparison of detrended fluctuation analysis and spectral analysis for heart rate variability in sleep and sleep apnea. IEEE Trans Biomed Eng. 2003;50(10):1143–51. https://doi.org/10.1109/TBME.2003.817636 PMID: 14560767
39. Mourot L, Bouhaddi M, Perrey S, Rouillon J-D, Regnard J. Quantitative Poincaré plot analysis of heart rate variability: effect of endurance training. Eur J Appl Physiol. 2004;91(1):79–87. https://doi.org/10.1007/s00421-003-0917-0 PMID: 12955518
40. Karmakar CK, Khandoker AH, Gubbi J, Palaniswami M. Complex correlation measure: a novel descriptor for Poincaré plot. Biomed Eng Online. 2009;8:17. https://doi.org/10.1186/1475-925X-8-17 PMID: 19674482
41. Webber CL Jr, Zbilut JP. Dynamical assessment of physiological systems and states using recurrence plot strategies. J Appl Physiol . 1994;76(2):965–73. https://doi.org/10.1152/jappl.1994.76.2.965 PMID: 8175612
42. Peng CK, Havlin S, Stanley HE, Goldberger AL. Quantification of scaling exponents and crossover phenomena in nonstationary heartbeat time series. Chaos: Interdiscip J Nonlinear Sci. 1995;5(1):82–7. https://doi.org/10.1063/1.166141 PMID: 11538314
43. Castiglioni P, Faini A. A fast DFA algorithm for multifractal multiscale analysis of physiological time series. Front Physiol. 2019;10:115. https://doi.org/10.3389/fphys.2019.00115 PMID: 30881308
44. Gronwald T, Hoos O, Hottenrott K. Effects of a short-term cycling interval session and active recovery on non-linear dynamics of cardiac autonomic activity in endurance trained cyclists. J Clin Med. 2019;8(2):194. https://doi.org/10.3390/jcm8020194 PMID: 30736284
45. Zhang S. Nearest neighbor selection for iteratively kNN imputation. J Syst Softw. 2012;85(11):2541–52. https://doi.org/10.1016/j.jss.2012.05.073
46. Su J, Beling PA, Guo R, Han K. Graph convolution networks for probabilistic modeling of driving acceleration. In: 2020 IEEE 23rd International Conference on Intelligent Transportation Systems (ITSC). IEEE; 2020. pp. 1–8. https://ieeexplore.ieee.org/document/9294294.
47. Ding Y, Fu M, Luo P, Wu F-X. Network learning for biomarker discovery. Int J Netw Dynam Intell. 2023:51–65. https://doi.org/10.53941/ijndi0201004
48. Hagberg A, Swart PJ, Schult DA. Exploring network structure, dynamics, and function using NetworkX. Los Alamos, NM (United States): Los Alamos National Laboratory (LANL); 2008. https://www.osti.gov/biblio/960616
49. Dai Q, Shen X, Zhang L, Li Q, Wang D. Adversarial training methods for network embedding. In: The World Wide Web Conference. 2019. 329–39. https://doi.org/10.1145/3308558.331344
50. Perrone G, Unpingco J, Lu HM. Network visualizations with Pyvis and VisJS. arXiv preprint arXiv:2006.04951. 2020. https://arxiv.org/abs/2006.04951
51. Fey M, Lenssen JE. Fast graph representation learning with PyTorch Geometric. arXiv preprint arXiv:1903.02428. 2019. https://arxiv.org/abs/1903.02428
52. Tritt A, Yue JK, Ferguson AR, Torres Espin A, Nelson LD, Yuh EL, et al. Data-driven distillation and precision prognosis in traumatic brain injury with interpretable machine learning. Sci Rep. 2023;13(1):21200. https://doi.org/10.1038/s41598-023-48054-z PMID: 38040784
53. Bhattacharyay S, Caruso PF, Åkerlund C, Wilson L, Stevens RD, Menon DK, et al. Mining the contribution of intensive care clinical course to outcome after traumatic brain injury. NPJ Digit Med. 2023;6(1):154. https://doi.org/10.1038/s41746-023-00895-8 PMID: 37604980
54. Åkerlund CAI, Holst A, Bhattacharyay S, Stocchetti N, Steyerberg E, Smielewski P, et al. Clinical descriptors of disease trajectories in patients with traumatic brain injury in the intensive care unit (CENTER-TBI): a multicentre observational cohort study. Lancet Neurol. 2024;23(1):71–80. https://doi.org/10.1016/S1474-4422(23)00358-7 PMID: 37977157

55. Billman GE. Heart rate variability - a historical perspective. Front Physiol. 2011;2:86. https://doi.org/10.3389/fphys.2011.00086 PMID: 22144961

56. Nakae R, Takayama Y, Kuwamoto K, Naoe Y, Sato H, Yokota H. Time course of coagulation and fibrinolytic parameters in patients with traumatic brain injury. J Neurotrauma. 2016;33(7):688–95. https://doi.org/10.1089/neu.2015.4039 PMID: 26414158

57. Papa L, Mendes ME, Braga CF. Mild traumatic brain injury among the geriatric population. Curr Transl Geriatr Exp Gerontol Rep. 2012;1(3):135–42. https://doi.org/10.1007/s13670-012-0019-0 PMID: 23589783

58. Munivenkatappa A, Agrawal A, Shukla DP, Kumaraswamy D, Devi BI. Traumatic brain injury: does gender influence outcomes? Int J Crit Illn Inj Sci. 2016;6(2):70–3. https://doi.org/10.4103/2229-5151.183024 PMID: 27308254

59. Machado C, Estevez M, Perez-Nellar J, Schiavi A. Residual vasomotor activity assessed by heart rate variability in a brain-dead case. BMJ Case Rep. 2015;2015. https://doi.org/10.1136/bcr-2014-205677 PMID: 25833905
